# Inequality in measuring scholarly success: Variation in the *h*-index within and between disciplines

**DOI:** 10.1371/journal.pone.0316913

**Published:** 2025-01-24

**Authors:** Ryan Light, Aaron Gullickson, Jill Ann Harrison

**Affiliations:** University of Oregon, Sociology, Eugene, OR, United States of America; Iuliu Hațieganu University of Medicine and Pharmacy: Universitatea de Medicina si Farmacie Iuliu Hatieganu, ROMANIA

## Abstract

Scholars and university administrators have a vested interest in building equitable valuation systems of academic work for both practical (e.g., resource distribution) and more lofty purposes (e.g., what constitutes “good” research). Well-established inequalities in science pose a difficult challenge to those interested in constructing a parsimonious and fair method for valuation as stratification occurs within academic disciplines, but also between them. The *h*-index, a popular research metric, has been formally used as one such method of valuation. In this article, we use the case of the *h*-index to examine how the distribution of research metrics reveal within and between discipline inequalities. Using bibliometric data from 1960-2019 on over 50,000 high performing scientists—the top 2% most frequently cited authors—across 174 disciplines, we construct random effects within-between models predicting the *h*-index. Results suggest significant within-discipline variation in several forms, specifically sole-authorship and female penalties. Results also show that a sole authorship penalty plays a significant role in well-known between-discipline variation. Field-specific models emphasize the “apples-to-oranges,” or incommensurable, property of cross-discipline comparison with significant heterogeneity in sole-authorship and female penalties within fields. In conclusion, we recommend continued caution when using the *h*-index or similar metrics for valuation purposes and the prioritization of substantive valuations from disciplinary experts.

## Introduction

From teaching to research, systematic evaluation of academic work presents a unique set of challenges as the academic disciplines that broadly organize scholarly labor differ by several relevant factors. Inequalities experienced by minoritized and women scholars point to processes of social closure or exclusionary practices that limit the opportunities for some scholars and not others across disciplines [1–3]. Cultural and economic differences between disciplines, like the prevalence of team science or even the definition of success, can affect the quality and quantity of publication [4–6]. Scholars have invented dozens of metrics that are formally or informally used in the evaluation of scholarly research; however, the risk of apples-to-oranges comparisons given within discipline and between discipline differences requires greater scrutiny [7, 8] alongside continued efforts towards metrics literacy, or the ability to “critically assess and effectively and ethically use scholarly metrics” [9]. Prior research identifies substantial variation in scholars’ understanding of metrics [10–12] as well as variation in materials for metrics literacy [9]. The most widely used metric of this type is the *h*-index, a simple score where a scholar with 10 published articles with at least 10 citations receives an *h*-index of 10. Academics use the *h*-index informally to assess impact via popular databases like Google Scholar, but also formally as listed in promotion policies and faculty handbooks [13]. The limitations of the *h*-index are well understood, but less is known about how processes of inequality are embedded both within and between disciplines. A greater understanding of these processes can help inform both formal and informal evaluation processes.

In this article, we examine within and between discipline variation in the *h*-index and its relationship to inequality and disciplinary culture at both levels. We focus our attention on gender inequality, both because prior research has demonstrated the multifaceted ways that gender inequality is produced within academic disciplines [14–18] and because gender identification of scholars is relatively straightforward in existing data sources due to gendered naming patterns. We examine the impact of disciplinary culture by focusing on the tendency for scholars to produce sole authored publications, which is a cultural practice that varies in likelihood across disciplines and fields and can affect overall citations metrics. Specifically, we ask three related questions:

1. What is the variation within and between disciplines in the *h*-index?
2. How do gender and sole authorship contribute to this variation at both levels?
3. How do these within and between discipline factors vary across fields (e.g., social sciences, medical sciences, natural sciences)?

Examining within and between discipline variation in the *h*-index offers insight into how metrics relate to inequalities and contributes to research on improving research metrics for evaluation purposes as well as research on the limitations of simplifying summaries of academic labor through quantification. While prior work examines the *h*-index by field, discipline, or gender, less is known about how gender and sole authorship contribute to *both* within *and* between discipline differences in *h*-index scores [19]. This study extends prior work on metrics in science generally and the *h*-index specifically by conceptualizing the inequality process as occurring via intra-disciplinary and inter-disciplinary dynamics. Variation in the *h*-index may result from “apples-to-oranges” comparisons across disciplines and fields due to specific factors, like gender and sole authorship, but also occur within disciplines, as scholars experience differing publishing trajectories dependent on these same factors.

### Gender inequality in science

Inequality in science, and in academia generally, persists within and between disciplines and along well-known axes. Gender is one of the most studied axes of inequality in science. Research on the scientific pipeline, for example, shows how obstacles, like cultural stereotypes about skills differences in math and science and related social psychological factors such as a sense of belonging, limit pathways to particular scientific fields for girls [20–22]. These pipeline factors continue throughout the research life course as men and women become segregated in doctoral programs by field and prestige [23]. When these significant obstacles are overcome, women continue to experience inequality in academic work, including in publication. Publication is both the outcome of academic labor and the currency of academic careers. How many and the type of publications produced by a scientist often translates into tangible resources, like salary raises and the job security of tenure, and less tangible resources, like prestige. While significant gains have been made by women in academic work—numerous fields that were male dominated in the mid- to late-twentieth century are now majority female—publication remains a potential site of stratification in terms of both the number of publications scholars accrue over their careers and the quality of those publications [3, 24]. Early signs indicate that these forms of gender inequality may have increased due to the COVID-19 pandemic or a “pandemic penalty” [25].

Sociologists of science have spent decades trying to disentangle the factors associated with gender differences in publication, especially related to differences in the number of publications and citations. Nearly 40 years ago, Cole and Zuckerman referred to the ongoing male advantage in publication and citation counts as the “productivity puzzle” because the causes of this advantage remained difficult to pinpoint [26]. They conclude, “[S]ince gender differences in published productivity persist, the productivity puzzle has yet to be solved” (pg. 250). Research suggests that the productivity puzzle remains. For example, Erin Leahey’s research shows how the level of research specialization interacts with gender to affect productivity, with consequences for earnings [27, 28]. More recent work provides evidence that the productivity puzzle in STEM fields results from variation between men and women in career length and exit rates as productivity appears to be more equal across shorter time horizons [29]. Scholars have also turned to more complex mechanisms that may help perpetuate gender and racial hierarchies in academic work. For example, using data on US doctoral recipients, Hofstra et. al. find that gender and racial minorities are more likely to generate innovative scientific work, but that this work is less likely to be adopted by future researchers with consequences for academic hiring [1].

A prestige puzzle may also coincide with the productivity puzzle as women may be less likely to publish in the most prestigious journals in their fields [30]. As top journals have higher impact factors, the prestige puzzle could have a significant impact on differences in publication metrics and career outcomes. This form of inequality may occur for a variety of reasons including a lack of mentorship, different family and work-based responsibilities between men and women, and differences in specialization similar to Leahey’s work on specialization and productivity [24, 27, 28]. Drawing on literature on occupational segregation and identity, this work on elite publication shows how the prestige puzzle has changed over time within sociology [24]. Earlier cohorts of women sociologists were significantly less likely to publish in top sociology journals compared to men regardless of specialty areas. However, as more women entered sociology, occupational segregation occurred with subfields becoming sharply gender imbalanced. While baseline models of the prestige puzzle for more recent cohorts reveal the persistence of this form of inequality, more complete models that control for specialization, or occupational identity, show that the contemporary effect likely operates through these segregation processes. More recent work on sociology, economics, and political science shows a null effect of gender on citation when social scientists are situated in similar disciplinary and sub-field spaces, suggesting that teasing apart the contexts when a gender penalty persists and when it does not remains an important concern for those interested in inequality [31].

Collaboration also likely plays a role with significant historical differences in coauthorship networks based on gender [32]. Recent research on computer scientists by Jadidi and coauthors finds significant differences in collaboration between men and women. Men are more likely to have larger coauthorship networks and to play brokerage roles within them, while women’s networks exhibit increasing gender homophily [33]. These differences relate to publication outcomes as the network factors positively affect measures of productivity, including the *h*-index. Jadidi and coauthors conclude that women “on average are less likely to adapt to the collaboration patterns that are related with success. However, those women who become successful computer scientists exhibit the same collaborative behavior as their successful male colleagues” (pg. 19). Research in both political science and sociology also identifies how team science affects gender and publication in these disciplines pointing to how the structure of scientific work can negatively impact women social scientists [34, 35].

Collaboration may impact individual-level inequalities beyond gender. Collaboration is broadly understood as a key aspect of epistemic culture. Epistemic culture consists of “those amalgams of arrangements and mechanisms…which, in a given field, make up how we know what we know” [36, pg. 1]. The questions that scholars ask and the strategies that they use to answer them differ by field with consequences for individuals embedded in specific cultures. Plainly, the structure of team science matters for publication outcomes. This suggests that within discipline differences may affect publication metrics, like the *h*-index, but also points to how inequality may occur between disciplines as pronounced differences exist between disciplines in terms of factors like gender composition and team versus sole authorship.

Together, prior research on inequality points to processes that may impact within and between discipline variation in the *h*-index. The *h*-index plays a role in academic hierarchies through informal and formal evaluation processes, but it is also shaped by broader factors structuring the academic landscape, like gender pipelines, epistemic culture, and so on.

### Inequality between disciplines and fields

Disciplinary differences affect inequality along several dimensions. For example, differences in gender composition may have direct and indirect effects on how resources are distributed in universities. Disciplinary cultures also differ and these differences may affect inequality. For example, disciplines differ in terms of how work is evaluated [37] or even how emotions are expressed at work [38]. Moreover, disciplines differ in terms of how academic work is conducted [29]. Do scholars collaborate in teams or are they more likely to work alone? Less is known about how these between discipline-based inequalities differ from within discipline inequality. Research on collaboration and citation impact using the *h*-index shows disciplinary differences in the effect that collaboration has on impact with more collaboration having a stronger positive effect in physics and medicine, while having a smaller effect in the brain sciences or computer science [39]. Disciplinary differences occur regarding more tangible resources, like federal funding. Research describes the substantial differences between fields in terms of federal funding with implications—a “domino effect”—for future non-federal funding and a potential site of cumulative advantage stratifying disciplines [40, 41].

One of the ways that disciplines differ and also one of the ways that disciplines may be valued differently is how they perform on commonly used metrics. Time-worn debates in the philosophy of science have tried to identify the implications of and/or reconcile the so-called “two cultures” or the division between the arts and the sciences [42]. While this debated formulation may exaggerate differences, the two cultures perspective draws attention to the variation that occurs across fields both at a philosophical-level, but also as a more practical concern. In terms of the latter, prior work has suggested field normalizing the *h*-index to account for between field variation [43]. Questions remain about the extent of variation within and between fields in addition to the variation occurring within and between disciplines situated in fields. Do field-level variations in gender composition and team science result in apple-to-oranges comparisons when using common scholarly metrics like the *h*-index?

### The risks of quantification

Quantification has become a central feature of contemporary life as “[a]dministration, management, and even mundane daily activities are increasingly structured around performance measures, cost-benefit analysis, risk calculations, ratings, and rankings” [44, p. 224]. Critiques often focus on the risks of quantification as a central factor in determining worth, which occurs in a variety of fields from the law to business to education [45, p. 4]. This “metrics fixation” is part of the broader process of neoliberalization of education. Neoliberalization succinctly captures an effort to “economize everything” [46, p. 171], such that neoliberal reason becomes common sense or simply the default rationale people use to make decisions. Metrics reinforce the notion that individual performance at work can be easily calculated and compared; therefore, material rewards like promotions and raises can be fairly and transparently applied. Of course, metrics often hide as much as they reveal as they simplify a process by carving away essential components. In universities, the metrics that help reinforce and are reinforced by neoliberal reason summarize entire careers for administrators who may have little understanding of the research that the metrics summarize.

University administrators’ use of metrics to evaluate faculty output is a fairly recent phenomenon. Prior to the development of bibliometric indicators, evaluation of scholarly research was performed primarily by disciplinary specialists who offered qualitative assessment of a research record. While peer assessment is still a central part of evaluation processes, metrics, such as citation counts, journal impact factors, and the *h*-index, are now commonly incorporated into hiring and promotion decisions [47] and are seen as more important to untenured scholars than tenured ones [48]. While many administrators likely have a broad understanding of such research metrics, such understanding may be informed by an administrator’s own disciplinary and professional background. Therefore, understanding how metrics relate to within and between differences across disciplines and fields requires greater scrutiny. Better understanding of such differences can help administrators avoid potential pitfalls in evaluation.

Critics have raised concerns about how metrics transform scholarship into a capitalist-like market at the core of neoliberalization resulting in “perverse incentives” for researchers to publish shoddy or fraudulent work and efforts to “game” the system at either the journal or individual-level [49, 50], while simultaneously resulting in mental health trauma for academic workers experiencing hypercompetitive markets and suspicious management [51]. From this perspective, administrators’ continued reliance upon metrics serves as a modern form of academic Taylorism, a production principle developed in the late nineteenth and early twentieth century that pursued technological solutions to the “problem” of worker-related inefficiencies on the shop floor with little regard to employee satisfaction or wellness [52]. By using technology to set the nature and pace of production, owners and managers gain greater control over the labor process itself. To critics, prioritizing metrics creates a demand for quantity over quality, and by following these demands academic laborers risk surrendering some degree of control over their own labor processes.

#### The case of the *h*-index

One key metric used for evaluation purposes is the *h*-index or Hirsch Index. Physicist Jorge Hirsch proposed the *h*-index as a “useful index to characterize the scientific output of a researcher” in a 2005 article in the Proceedings of the National Academy of Sciences [53]. While acknowledging the “potentially distasteful” use of metrics for evaluation, he presents quantification as an economical means of evaluating impact. In this highly cited article, Hirsch defines the *h*-index as follows: “A scientist has index *h* if *h* of his or her *N*_*p*_ papers have at least *h* citations each and the other (*N*_*p*_ − *h*) papers have ≤*h* citations each” (p. 16569). In other words, a scholar with 10 of their 100 publications with a citation count of 10 or higher will have an *h*-index of 10. He goes on to specify—again with some acknowledgement that metrics offer a “rough approximation” of a research portfolio—how and when the index could be put to use: “Based on typical *h* and *m* values found, I suggest (with large error bars) that for faculty at major research universities, *h* ≈ 12 might be a typical value for advancement to tenure (associate professor) and that *h* ≈ 18 might be a typical value for advancement to full professor” (p. 16571). In sum, this publication announced a simple means of evaluating research impact and permission to use the metric for evaluation purposes.

The immediate response to the *h*-index was largely positive with features in top scientific journals; however, some criticism of the index also quickly appeared [54]. Critics identified a range of issues from the relationship between the *h*-index and career length as well as the effect of self-citation [55, 56, among others]. However, the *h*-index and variants have proven enormously popular both in the bibliometrics and science of science communities and among university administrators seeking quick and cheap ways to evaluate scholars, including universities and science funding agencies [54]. The *h*-index is included as a key quantitative metric for annual review and/or tenure and promotion in faculty handbooks in a range of departments and schools in the United States (c.f., handbooks from the Boston University School of Public Health [57], the Ohio State University Department of Surgery [58] or Oregon State University’s College of Business [59]). Survey research in Germany on whether and how scholars understand the importance of the *h*-index indicates that natural scientists widely understand the importance of the *h*-index to their careers, but scholars in the humanities and social sciences do not [11]. This variation in knowledge motivates calls for expanding metrics literacy efforts [9, 12, 60]. In sum, despite some criticism, the *h*-index has been widely adopted although perhaps not widely understood. This analysis contributes to the broader literature on metrics and science by examining the factors contributing to within and between discipline variation in the *h*-index.

### Hypotheses

We develop the following hypotheses to better understand within and between discipline differences in the *h*-index, or Hirsch Index, based on the prior literature. Consistent with work on gender inequality and science and particularly work on the productivity and prestige puzzles, we examine the following:

#### Within discipline hypotheses

In light of research on team science and its impact on academic careers, we examine the following:

*Female Penalty Hypothesis (H1):* Authors with names more common among women will have lower *h*-index scores, on average.

*Sole Author Hypothesis (H2):* Authors with a higher share of sole-authored publications will have lower *h*-index scores, on average.

#### Between disciplines hypotheses

Although less well understood, based on the research describing disciplinary differences in culture, such as propensity to collaborate, and material differences in resources, we examine two related hypotheses:

*Feminized Discipline Hypothesis (H3a):* Disciplines with a higher share of women will have lower *h*-index scores, on average.

*Teamwork Variation Hypothesis (H3b):* Disciplines with a higher share of sole authorship will have lower *h*-index scores, on average.

#### Field-level hypothesis

Finally, a field-level view may help disentangle cultural and compositional effects and shed light on the question of “apples-to-oranges” comparison within and between fields. We, therefore, evaluate the following hypothesis:

*Field Variation Hypothesis (H4):* Significant field differences will exist in the relationship of the *h*-index to gender and sole authorship at both the individual and disciplinary level.

## Materials and methods

To evaluate these hypotheses, we analyze data on high performing scholars according to well-known metrics. Ioannidis and coauthors [8] collected author-level bibliometrics on 100,000 high performing scholars from the Scopus database. They updated this data through 2019 and expanded to include the top 2% of authors overall across a wide range of disciplines [61]. These data suffer from several limitations, including coverage differences between disciplines within the Scopus database [62, 63]. Nonetheless, these well-curated data represent a unique opportunity to evaluate within and between disciplinary differences in *h*-index scores. We also see these data as offering a conservative test of such differences as variation is likely to widen when moving beyond scholars who are in the top 2% on these metrics.

We reduce the data along several dimensions to address some of the limitations of the Scopus database and for analytic purposes. First, we limit the analysis only to the 68,016 scholars in the United States to account for geographic variation in the Scopus database and geographic variation in how universities are structured. We then further restrict the analytical data to those who first published in 1960 or after and those who last published in 2017 or later to identify active scholars and reduce the impact of historical, rather than contemporary, patterns. These reductions, along with a small number of missing values on the variables below, leave us a final analytical sample size of 54,825 scholars nested in 174 disciplines.

The dependent variable for the analysis is the *h*-index. The key independent variables are the probability of female name and the percentage of sole authored publications. We also include additional control variables of the count of scholars from the same university in the same dataset to measure highly productive university environments, a specialization score measured as the proportion of total articles appearing in the main discipline for each author, and career length, measured by the years between each scholar’s first and most recent publication.

Estimating gender is problematic for numerous reasons including the typical reliance on government-provided data that often assumes and contributes to the gender binary [64], and these methods should be used with caution and only when necessary. In this case, names are our only means of estimating gender. Here, we draw on first name data from the 1940–1990 US Social Security Administration to assign a probability of female name using the gender package in R [65]. We use this probability directly in our models, rather than assigning an arbitrary cutoff for a binary gender assignment. A substantial number of cases (18%) are missing on this variable, usually because they were identified by initials rather than a full first name. Rather than lose this many cases, we use multiple imputation to assign values to the probability of female name based on the respondent’s other variables, including discipline. We impute five complete datasets and pool analyses across these datasets for all of the models presented here.

We also consider how the patterns we observe may differ within large fields among disciplines. To explore this issue, we divide the total 174 disciplines into five large fields of the humanities, medical (including public health), professional, social sciences (including policy), and science, technology, engineering, and math (STEM). These fields differ from the fields provided in the original data but more closely correspond to the division of disciplines within the American academy. The supplementary materials show a full list of disciplines, which disciplines were assigned to each field, and summary statistics for each discipline.

Table 1 presents the descriptive statistics for all variables used in the analyses for the total sample and across the five different fields.

**Table 1:** Mean and standard deviation on key variables for the whole sample and by field, based on first complete dataset.

Variable	All	Fields				
		Humanities	Medical	Prof.	Soc. Sci.	STEM
h-index	44.1 (20.9)	19.8 (10.4)	52.2 (21.7)	29.4 (10.7)	33.1 (15.0)	38.8 (17.6)
% female	19.0 (37.6)	30.6 (45.0)	22.0 (40.0)	21.4 (39.5)	27.8 (43.6)	13.5 (32.0)
% sole author	10.7 (14.4)	56.0 (31.4)	8.1 (9.4)	18.2 (16.8)	24.6 (22.9)	9.1 (11.5)
career length	35.3 (10.1)	33.6 (11.2)	36.2 (9.3)	32.2 (9.9)	34.7 (10.6)	34.7 (10.6)
specialization	0.73 (0.19)	0.64 (0.21)	0.76 (0.18)	0.75 (0.21)	0.72 (0.19)	0.70 (0.20)
uni. count	198 (202)	255 (204)	185 (201)	166 (203)	246 (209)	204 (200)
N	54,825	856	25,581	1,228	3,987	23,173

Note: standard deviations shown in parenthesis

### Analytic strategy

We model variation in *h*-index scores across scholars using random effects within-between (REWB) models [66]. Random effects within-between (REWB) models are a variant of multilevel models that allows researchers to estimate the effect of a given predictor variable both within (as per a standard “fixed effects” model) and between the higher level clusters (in this case disciplines). In general, the structure of the REWB model is:

$$\begin{array}{c} y_{ij}=\beta_{0j}+\beta_{1}\left(x_{ij}-{\bar{x}}_{.j}\right)+\beta_{2}\left({\bar{x}}_{.j}\right)+\upsilon_{0j}+\epsilon_{ij} \end{array}$$

Where *y*_*ij*_ is the outcome for the *i*th unit in the *j*th cluster and *x*_*ij*_ is the predictor variable for the *i*th unit in the *j*th cluster. *υ*_0*j*_ and *ϵ*_*ij*_ are cluster-level and individual-level random errors, respectively. Because the mean of *x* for cluster *j* (${\bar{x}}_{.j}$) is included in the model and *x*_*ij*_ values are mean centered by cluster, the *β*_1_ parameter is identical to that of a fixed-effects model in which all between variance is absorbed by cluster-level dummy variables. However, the REWB model also includes an estimate of the between cluster effect of *x* estimated in *β*_2_, which is impossible in a fixed-effects model. This *β*_2_ term is equivalent to the estimate obtained by aggregating data to the higher level and examining the relationship between the means of *x* and *y*. Thus, this model maintains the advantage of traditional fixed effects models by absorbing all cluster level differences when estimating lower level parameters, while at the same time allowing for an analysis of the “contextual” relationships at the higher level.

We use this feature to estimate both within and between effects of gender and sole authorship on a scholar’s *h*-index. Both the probability of a female name variable and the sole authorship variable are mean centered by discipline at the individual level so that the interpretation of their effect is solely among scholars within the same discipline. For the between discipline effects, we calculate the mean probability of a female name and the mean sole authorship of each discipline. For probability of a female name, we use a 0 to 100 percentage scale at the disciplinary level for ease of interpretation of coefficients as the expected change in the *h*-index for a one percentage point increase in the mean probability of a female name. For sole authorship, we use a natural log transformation of the mean percent sole-authorship variable at the disciplinary level because exploratory analysis indicated a negative diminishing returns relationship between sole-authorship and the *h*-index at this level.

The control variables of career length, specialization, and university publication count are only included as grand mean centered individual level variables. We also standardize university publication count to have a mean of zero and a standard deviation of one for ease of interpretation.

In addition to the models for the full data, we also run these same models separately for each of these fields to explore differences in patterns across fields.

## Results

We begin by analyzing a partition of the variance in the *h*-index within and between disciplines in a null multilevel model. The percentage of the total variation in the *h*-index that occurs between disciplines is given by the intraclass correlation coefficient (ICC) of this model. Table 2 shows the ICC for the model across all observations as well as separately by field. In total, roughly a third of the variation in the *h*-index occurs between disciplines.

**Table 2:** Partition of variance in *h*-index scores between and within disciplines.

Grouping	All (%)	Field				
		Humanities (%)	Medical (%)	Prof. (%)	Soc. Sci. (%)	STEM (%)
Between discipline	32.0	53.6	20.4	16.2	31.3	20.1
Within discipline	68.0	46.4	79.6	83.8	68.7	79.9

The ICC is substantial across all fields as well, but also varies substantially. Slightly more than half the variation in the *h*-index among scholars in the humanities is between disciplines, while only one-fifth or less of the variation in the *h*-index is between disciplines among scholars in the medical, STEM, and professional fields.

Table 3 presents the multilevel models predicting the *h*-index across all disciplines. Model 1 predicts *h*-index scores by the individual and disciplinary variables for gender. Model 2 predicts *h*-index scores by the individual and disciplinary variables for sole authorship. Model 3 includes both sets of predictor variables from Models 1 and 2 together. Finally, Model 4 includes additional control variables for career length, specialization, and highly productive universities. The estimates for the key predictor variables of gender and sole-authorship are robust to these additional controls, although gender differences do decline in size somewhat. We use the results from Model 4 to describe overall patterns below, except where noted otherwise.

**Table 3:** Multilevel models predicting *h*-index score across all disciplines with clustering at the disciplinary level.

	Model 1	Model 2	Model 3	Model 4
Intercept	39.32*	69.64*	68.86*	68.91*
	(1.80)	(2.12)	(2.13)	(2.06)
Prob. [0–1] of female name†	−3.00*		−3.67*	−2.63*
	(0.23)		(0.22)	(0.21)
Disc. mean percent female name	−0.15*		0.10*	0.11*
	(0.07)		(0.05)	(0.05)
Percent sole authored pubs†		−0.41*	−0.42*	−0.47*
		(0.01)	(0.01)	(0.01)
Disc. mean percent sole authored (log)		−12.87*	−13.39*	−13.53*
		(0.79)	(0.82)	(0.79)
Career length*				0.35*
				(0.01)
Specialization [0–1]*				−2.66*
				(0.44)
Highly productive uni. count (stdized)*				1.72*
				(0.07)
Residual SD between discipline	11.86	7.41	7.33	7.05
Residual SD within discipline	17.41	16.83	16.78	16.34
N (discipline)	174	174	174	174
N (individual)	54825	54825	54825	54825

*p < 0.05.

Standard errors shown in parenthesis.

^†^=discipline mean centered;

*=grand mean centered.

Consistent with the productivity puzzle, female scholars have an *h*-index approximately 2.63 points lower than male scholars in the same discipline, on average. More frequent sole authorship is also associated with a lower *h*-index. Within the same discipline, a one percentage point increase in the percent of sole-authored publications for a given scholar is associated with a 0.47 lower *h*-index score, on average.

Additionally, we find substantial differences between disciplines in *h*-index scores based on the feminization of the discipline and sole-authorship norms. Sole authorship behaves as expected. A one percent increase in the mean percent sole-authored publications in a discipline is associated with a 0.135 decline in the mean *h*-index for that discipline. Thus, cultural norms of more sole-authorship within a discipline contribute to lower overall *h*-index scores for that discipline.

The feminization of a discipline, as indicated by the mean percent female name within the discipline, has a more complex relationship to *h*-index scores. Model 1 shows a slightly negative association between percent female and mean *h*-index scores across disciplines. However, this slightly negative association is a spurious byproduct of the underlying tendency for more feminized disciplines to also be more focused on sole authorship (*r* = 0.30). Model 3 shows that, once we hold constant the disciplinary tendency for sole-authorship, the association between feminization and *h*-index scores becomes slightly positive. In the final model, we estimate that a one percentage point increase in the percent female name within a discipline is associated with a 0.11 increase in the mean *h*-index score for that discipline. Thus, when we compare disciplines with similar sole-authorship tendencies, feminized disciplines actually have a slight advantage in mean *h*-index scores, even though, within disciplines, women are still disadvantaged relative to men.

Table 4 presents models equivalent to Model 4 in Table 3, but separated by field. The most notable change in these sets of models is that there is no observable effect of disciplinary feminization within all fields, except for the Social Sciences. In contrast, we observed a moderate positive effect of disciplinary feminization when we pool all fields. This finding implies that the slight positive effect of disciplinary feminization was driven by compositional issues between fields and differences in overall field productivity. The remaining variables are consistent in direction, but vary substantially in magnitude across fields. The within-discipline differences by gender are smallest in the humanities and STEM fields and largest in the medical field. Similarly, the within-discipline differences by sole-authorship are smallest in the humanities and largest in the medical field. The between-discipline effect of sole-authorship is largest in the medical field and smallest in the professional field where it is only a third as large.

**Table 4:** Multilevel models predicting *h*-index score within each field. Clustering at the disciplinary level.

	Humanities	Medical	Prof.	Soc. Sci.	STEM
Intercept	79.45*	93.04*	47.21*	77.81*	58.73*
	(12.68)	(6.37)	(8.23)	(4.57)	(5.18)
Prob. [0–1] of female name†	−1.30*	−3.73*	−1.57*	−2.11*	−1.25*
	(0.50)	(0.31)	(0.76)	(0.43)	(0.34)
Disc. mean percent female name	−0.02	0.07	−0.01	−0.13*	0.12
	(0.09)	(0.08)	(0.21)	(0.05)	(0.17)
Percent sole authored pubs†	−0.16*	−0.66*	−0.26*	−0.24*	−0.49*
	(0.01)	(0.01)	(0.02)	(0.01)	(0.01)
Disc. mean percent sole authored (log)	−15.38*	−21.79*	−6.84*	−13.66*	−10.57*
	(3.08)	(3.02)	(3.34)	(1.19)	(1.83)
Career length*	0.13*	0.45*	0.29*	0.31*	0.30*
	(0.02)	(0.01)	(0.03)	(0.02)	(0.01)
Specialization [0–1]*	−3.13*	0.03	−0.46	−6.31*	−4.36*
	(1.10)	(0.79)	(1.55)	(1.03)	(0.57)
Highly productive uni. count (stdized)*	0.55*	1.51*	0.60*	0.38*	2.16*
	(0.21)	(0.12)	(0.29)	(0.18)	(0.10)
Residual SD between discipline	4.51	6.70	4.27	3.13	6.22
Residual SD within discipline	5.98	18.69	9.48	11.31	14.41
N (discipline)	15	52	12	28	67
N (individual)	856	25581	1228	3987	23173

*p < 0.05.

Standard errors shown in parenthesis.

^†^=discipline mean centered;

*=grand mean centered.

## Discussion

The substantial heterogeneity across disciplines (roughly a third of all variation) indicates that comparing the *h*-index across disciplines is problematic because of overall differences in culture, productivity, and citation patterns across disciplines. Additionally, we found that fields vary in the level of disciplinary heterogeneity. Some fields, like the medical, STEM, and professional fields, seem to have more shared culture and practices in terms of publishing productivity, while in other fields, we observe substantial disciplinary heterogeneity. Scholars in fields with greater commonality may misperceive the comparability of the *h*-index across disciplines more broadly.

Results indicate that, within disciplines, gender and sole authorship affect the *h*-index. Consistent with H1 and H2, we find that women have lower *h*-index scores than men and that more sole-authorship is associated with lower *h*-index scores, respectively. Thus, among scholars in the same discipline, variation in both ascriptive characteristics such as gender and research practices can generate substantial inequalities in *h*-index scores.

We find evidence of between discipline differences as well. Disciplines with more overall sole authorship have substantially lower mean *h*-index scores overall, consistent with the teamwork variation hypothesis (H3b). Support for the feminized discipline hypothesis (H3a) is more mixed. In the full models, results vary by whether disciplinary sole-authorship practice is included in the model because feminized disciplines are more likely to be high sole-authorship disciplines as well. In field specific models, we find a negative feminization effect on *h*-index scores only for the Social Sciences, and on effect for the remaining disciplines, even when controlling for disciplinary sole authorship. Thus, to the extent, that the feminization effect exists, it appears limited to certain fields.

We also observe that both the within discipline and between discipline effects vary substantially across fields, consistent with the field variation hypothesis (H4). This heterogeneity in models across fields implies an additional apples-to-oranges comparison problem when comparing scholars across fields. Even if some attention is paid to the overall predictors of productivity within and between disciplines when evaluating scholars, field heterogeneity in the effects of these predictors will complicate the ability to make accurate comparisons.

Several limitations suggest avenues for future research. First, the data select on high-performing scholars, and, therefore do not generalize to the broader population of academics. While we believe that this limitation likely results in a conservative estimate of within and between discipline inequality, more data on academia writ large are required to verify this claim. Second, the estimation of gender suffers from well-known limitations. Data linking self-reported gender beyond the gender binary to citation data would be a welcome resource. Gender estimation also required reducing the dataset by country. Third, and along similar lines, most scholarship on publication and inequality focuses on gender because it is possible to infer gender from names, despite some weaknesses in the approach. Other aspects of inequality, like race, class, ethnicity, sexual orientation, and whether one is a foreign-born academic or not remain under-studied. Future research would benefit from data linking self-reports of these characteristics to large bibliometric data to better understand the broad effects of inequality in academic work.

## Conclusion

This analysis provides further evidence that metrics in performance evaluation in academia should be used with caution at minimum and efforts towards metrics literacy should include both within and between discipline variation. Metrics are subject to within and between discipline biases that hinder their value in making both intradisciplinary and interdisciplinary comparisons. Of course, these comparisons are exactly what the quantification of scholarly work proposes to facilitate. Equitable evaluation should prioritize substantive assessment by disciplinary experts and/or content specialists. Like other forms of quantification, academic metrics simplify complex processes at a cost. This cost can reinforce existing inequalities and even generate new “automated inequalities” [67]. Those using metrics for evaluation, as Koopman and Galton [68] write about data usage generally, “need to be fervently attentive to the ways in which inequalities may be designed into their data” (pg. 16). In this vein, this analysis provides evidence of within and between discipline differences in the *h*-index with a focus on gender and sole authorship, but variation in scholarly metrics may extend to other dimensions of power, including race, class, and sexual orientation, or be related to other academic practices, like research funding, teaching loads, or service responsibilities.

## Supporting information

S1 Table Descriptive statistics by discipline. (PDF)
