# Supplementary material for: Inequality in measuring scholarly success: Variation in the h-index within and between disciplines
Source: PLoS One. 2025 Jan 24;20(1):e0316913. doi: 10.1371/journal.pone.0316913 (PMC11760043; doi:10.1371/journal.pone.0316913)
Supplement: S1 Table — (PDF) [file pone.0316913.s001.pdf]

Supplementary Materials

Ryan Light Aaron Gullickson Jill Ann Harrison

Table 1: Discipline statistics and mean values on variables, part 1

discipline	field	n	h-index	female	sole auth.
Anthropology	Humanities	104	29.7	33.1%	41.5%
Applied Ethics	Humanities	67	30.9	23.5%	36.1%
Archaeology	Humanities	87	24.8	25.1%	30.6%
Art Practice, History & Theory	Humanities	15	8.0	53.0%	90.3%
Classics	Humanities	14	11.9	13.0%	92.0%
Communication & Media Studies	Humanities	117	25.5	36.2%	26.4%
Drama & Theater	Humanities	6	8.0	33.4%	78.5%
Folklore	Humanities	5	10.6	20.2%	50.4%
History	Humanities	65	11.1	22.7%	77.6%
History of Science, Technology & Medicine	Humanities	5	15.6	60.0%	57.1%
Languages & Linguistics	Humanities	82	20.2	54.1%	54.3%
Literary Studies	Humanities	100	9.7	40.5%	86.5%
Music	Humanities	21	12.5	19.5%	54.7%
Philosophy	Humanities	100	17.2	16.8%	82.1%
Religions & Theology	Humanities	68	13.3	16.6%	63.1%
Allergy	Medical	118	51.9	20.3%	11.7%
Anatomy & Morphology	Medical	34	27.7	37.4%	11.2%
Anesthesiology	Medical	284	40.2	15.9%	10.7%
Arthritis & Rheumatology	Medical	212	57.4	30.8%	8.9%
Biochemistry & Molecular Biology	Medical	1325	54.3	17.5%	5.8%
Biophysics	Medical	133	46.2	15.9%	8.4%
Cardiovascular System & Hematology	Medical	1380	62.3	16.2%	7.6%
Complementary & Alternative Medicine	Medical	55	22.2	32.8%	26.8%
Dentistry	Medical	320	36.1	14.0%	13.9%
Dermatology & Venereal Diseases	Medical	255	46.9	26.2%	11.7%
Developmental Biology	Medical	1597	59.3	21.8%	6.7%
Emergency & Critical Care Medicine	Medical	255	45.4	9.6%	9.1%
Endocrinology & Metabolism	Medical	847	56.8	26.4%	7.1%
Environmental & Occupational Health	Medical	81	34.4	32.4%	11.1%
Epidemiology	Medical	133	63.8	35.4%	5.7%
Gastroenterology & Hepatology	Medical	524	53.7	13.4%	9.9%
General & Internal Medicine	Medical	643	40.4	25.9%	13.9%
General Clinical Medicine	Medical	61	29.4	11.1%	14.0%
Genetics & Heredity	Medical	265	58.5	33.1%	5.7%
Geriatrics	Medical	76	54.7	32.0%	10.3%
Gerontology	Medical	102	46.7	46.9%	13.2%

Health Policy & Services	Medical	176	43.6	23.6%	12.4%
Immunology	Medical	1186	62.9	20.7%	5.4%
Legal & Forensic Medicine	Medical	28	24.4	7.0%	14.8%
Microbiology	Medical	1028	52.6	21.9%	6.4%
Microscopy	Medical	26	37.7	5.1%	9.7%
Mycology & Parasitology	Medical	79	37.9	15.6%	7.2%
Neurology & Neurosurgery	Medical	2849	56.1	20.4%	7.7%
Nuclear Medicine & Medical Imaging	Medical	842	44.8	17.7%	7.0%
Nursing	Medical	318	27.7	87.4%	23.2%
Nutrition & Dietetics	Medical	251	49.1	40.0%	9.7%
Obstetrics & Reproductive Medicine	Medical	461	46.4	29.0%	9.3%
Oncology & Carcinogenesis	Medical	2155	61.5	24.2%	5.4%
Ophthalmology & Optometry	Medical	529	45.3	18.6%	8.7%
Orthopedics	Medical	519	45.0	6.3%	9.2%
Otorhinolaryngology	Medical	308	35.7	17.8%	9.2%
Pathology	Medical	198	54.8	24.8%	6.7%
Pediatrics	Medical	480	41.2	26.5%	9.9%
Pharmacology & Pharmacy	Medical	475	40.5	17.2%	7.6%
Physiology	Medical	193	44.2	11.7%	9.2%
Psychiatry	Medical	751	58.9	26.0%	8.5%
Public Health	Medical	530	46.9	39.8%	8.5%
Rehabilitation	Medical	160	36.9	36.7%	10.3%
Respiratory System	Medical	428	54.8	10.7%	8.4%
Speech-Language Pathology & Audiology	Medical	94	32.4	46.3%	13.6%
Sport Sciences	Medical	136	39.1	14.9%	10.0%
Substance Abuse	Medical	254	47.7	27.3%	7.7%
Surgery	Medical	737	47.8	13.8%	8.8%
Toxicology	Medical	385	43.8	24.5%	7.4%
Tropical Medicine	Medical	151	39.5	17.8%	6.3%
Urology & Nephrology	Medical	591	52.5	11.8%	10.6%
Virology	Medical	563	55.7	21.8%	5.1%
Accounting	Professional	44	24.1	27.4%	22.0%
Architecture	Professional	8	9.9	32.0%	37.3%
Building & Construction	Professional	105	29.9	8.1%	10.1%
Business & Management	Professional	460	31.6	22.9%	18.9%
Design Practice & Management	Professional	76	28.2	14.4%	10.1%
Development Studies	Professional	22	27.9	37.0%	33.8%
Finance	Professional	127	27.0	8.7%	21.9%
Industrial Relations	Professional	9	18.3	33.6%	39.4%
Logistics & Transportation	Professional	159	28.4	23.5%	11.7%
Marketing	Professional	124	31.1	28.8%	15.6%
Sport, Leisure & Tourism	Professional	41	29.8	36.6%	17.7%
Urban & Regional Planning	Professional	53	25.8	24.9%	41.3%
Agricultural Economics & Policy	Social Sciences	63	32.4	16.9%	19.3%
Behavioral Science & Comparative Psychology	Social Sciences	154	37.1	33.6%	17.6%
Clinical Psychology	Social Sciences	177	46.4	24.5%	12.5%
Criminology	Social Sciences	124	34.7	26.1%	19.8%
Cultural Studies	Social Sciences	38	13.7	35.0%	80.9%
Demography	Social Sciences	26	31.5	34.8%	27.6%
Developmental & Child Psychology	Social Sciences	402	45.2	42.4%	11.1%
Econometrics	Social Sciences	39	33.1	5.6%	22.5%
Economic Theory	Social Sciences	13	26.2	8.3%	34.6%
Economics	Social Sciences	459	30.2	13.2%	31.5%

Education	Social Sciences	552	25.1	36.6%	25.1%
Experimental Psychology	Social Sciences	480	41.3	29.7%	14.0%
Family Studies	Social Sciences	42	33.0	43.0%	16.4%
Gender Studies	Social Sciences	13	18.3	63.1%	57.3%
General Psychology & Cognitive Sciences	Social Sciences	44	29.2	42.0%	16.3%
Geography	Social Sciences	82	27.7	20.9%	39.6%
History of Social Sciences	Social Sciences	19	19.5	5.8%	47.9%
Human Factors	Social Sciences	141	35.5	31.3%	10.3%
Information & Library Sciences	Social Sciences	75	20.0	42.6%	31.7%
International Relations	Social Sciences	60	20.6	15.2%	59.7%
Law	Social Sciences	113	17.3	13.3%	63.0%
Political Science & Public Administration	Social Sciences	186	26.2	13.7%	38.8%
Psychoanalysis	Social Sciences	28	20.0	23.6%	68.3%
Science Studies	Social Sciences	20	26.2	10.9%	49.9%
Social Psychology	Social Sciences	371	40.2	26.4%	15.1%
Social Sciences Methods	Social Sciences	57	31.9	12.6%	23.1%
Social Work	Social Sciences	69	22.3	53.5%	23.3%
Sociology	Social Sciences	140	26.2	22.6%	39.8%
Acoustics	STEM	212	31.4	11.6%	11.6%
Aerospace & Aeronautics	STEM	551	24.4	8.5%	11.2%
Agronomy & Agriculture	STEM	312	35.2	15.8%	6.0%
Analytical Chemistry	STEM	374	41.7	16.9%	6.3%
Applied Mathematics	STEM	113	37.8	11.3%	15.1%
Applied Physics	STEM	1198	44.7	10.5%	6.1%
Artificial Intelligence & Image Processing	STEM	1160	33.0	16.1%	9.8%
Astronomy & Astrophysics	STEM	759	54.9	10.8%	7.1%
Automobile Design & Engineering	STEM	1	13.0	0.4%	7.1%
Bioinformatics	STEM	171	45.6	18.0%	7.1%
Biomedical Engineering	STEM	416	44.6	18.2%	4.9%
Biotechnology	STEM	234	41.5	16.7%	6.0%
Chemical Engineering	STEM	228	38.3	8.2%	6.8%
Chemical Physics	STEM	638	51.0	12.3%	7.2%
Civil Engineering	STEM	221	25.5	11.2%	12.5%
Computation Theory & Mathematics	STEM	136	34.6	11.9%	14.8%
Computer Hardware & Architecture	STEM	199	33.7	9.3%	6.2%
Dairy & Animal Science	STEM	258	34.0	8.8%	5.9%
Distributed Computing	STEM	116	29.3	9.1%	7.6%
Ecology	STEM	677	42.8	18.2%	10.8%
Electrical & Electronic Engineering	STEM	355	28.9	10.1%	8.4%
Energy	STEM	888	31.9	11.0%	8.8%
Entomology	STEM	191	34.8	14.4%	10.0%
Environmental Engineering	STEM	288	35.8	9.9%	9.4%
Environmental Sciences	STEM	355	37.4	15.5%	8.1%
Evolutionary Biology	STEM	353	42.0	15.6%	12.9%
Fisheries	STEM	160	34.0	7.6%	7.5%
Fluids & Plasmas	STEM	336	42.7	7.8%	11.5%
Food Science	STEM	174	37.4	17.0%	8.1%
Forestry	STEM	148	28.4	11.4%	7.5%
General Chemistry	STEM	222	50.2	18.6%	3.5%
General Mathematics	STEM	330	25.1	7.6%	30.1%
General Physics	STEM	302	39.9	12.2%	11.5%
Geochemistry & Geophysics	STEM	698	42.7	10.2%	10.3%
Geological & Geomatics Engineering	STEM	292	33.3	15.2%	11.4%

Geology	STEM	45	32.8	11.7%	17.1%
Horticulture	STEM	51	25.9	23.1%	10.7%
Industrial Engineering & Automation	STEM	503	34.3	10.4%	8.4%
Information Systems	STEM	180	33.1	22.2%	14.6%
Inorganic & Nuclear Chemistry	STEM	175	47.1	13.6%	4.3%
Marine Biology & Hydrobiology	STEM	265	42.2	16.4%	9.0%
Materials	STEM	713	37.9	15.2%	7.2%
Mathematical Physics	STEM	15	31.7	0.4%	19.9%
Mechanical Engineering & Transports	STEM	484	31.2	9.8%	12.7%
Medical Informatics	STEM	104	33.2	18.4%	9.8%
Medicinal & Biomolecular Chemistry	STEM	419	38.2	18.5%	4.9%
Meteorology & Atmospheric Sciences	STEM	814	47.5	12.0%	8.0%
Mining & Metallurgy	STEM	43	21.7	8.4%	11.4%
Nanoscience & Nanotechnology	STEM	449	57.2	23.3%	3.1%
Networking & Telecommunications	STEM	1153	31.5	13.8%	8.2%
Nuclear & Particle Physics	STEM	666	49.1	8.4%	13.1%
Numerical & Computational Mathematics	STEM	94	29.6	7.8%	18.5%
Oceanography	STEM	146	38.6	10.2%	11.9%
Operations Research	STEM	168	31.8	9.6%	16.2%
Optics	STEM	388	40.6	13.6%	7.1%
Optoelectronics & Photonics	STEM	636	29.7	9.9%	8.4%
Organic Chemistry	STEM	421	49.5	10.2%	5.0%
Ornithology	STEM	26	30.3	7.9%	11.8%
Paleontology	STEM	154	39.8	16.8%	14.5%
Physical Chemistry	STEM	69	50.4	17.9%	4.7%
Plant Biology & Botany	STEM	571	43.0	23.2%	6.3%
Polymers	STEM	335	45.5	11.5%	6.3%
Software Engineering	STEM	197	34.0	18.9%	10.8%
Statistics & Probability	STEM	196	39.6	11.9%	14.4%
Strategic, Defence & Security Studies	STEM	148	24.9	15.5%	27.4%
Veterinary Sciences	STEM	393	32.5	28.5%	8.8%
Zoology	STEM	86	24.7	12.3%	15.0%

Table 2: Discipline statistics and mean values on variables, part 2

discipline	field	career len.	spec.	uni. count
Anthropology	Humanities	40.4	0.546	279.5
Applied Ethics	Humanities	36.9	0.480	172.0
Archaeology	Humanities	33.7	0.740	246.9
Art Practice, History & Theory	Humanities	29.5	0.584	280.7
Classics	Humanities	34.0	0.731	360.8
Communication & Media Studies	Humanities	29.8	0.596	277.5
Drama & Theater	Humanities	35.3	0.590	247.5
Folklore	Humanities	42.8	0.463	226.2
History	Humanities	36.0	0.645	228.7
History of Science, Technology & Medicine	Humanities	38.6	0.566	202.4
Languages & Linguistics	Humanities	32.5	0.760	283.3
Literary Studies	Humanities	27.3	0.687	290.1
Music	Humanities	31.6	0.719	258.2
Philosophy	Humanities	36.3	0.708	271.6
Religions & Theology	Humanities	30.9	0.616	168.1

Allergy	Medical	38.4	0.904	154.7
Anatomy & Morphology	Medical	32.0	0.521	105.2
Anesthesiology	Medical	33.8	0.901	195.1
Arthritis & Rheumatology	Medical	36.7	0.880	184.3
Biochemistry & Molecular Biology	Medical	40.1	0.655	176.9
Biophysics	Medical	38.1	0.628	216.7
Cardiovascular System & Hematology	Medical	36.7	0.848	175.8
Complementary & Alternative Medicine	Medical	30.1	0.773	138.3
Dentistry	Medical	38.0	0.859	187.8
Dermatology & Venereal Diseases	Medical	37.5	0.884	177.2
Developmental Biology	Medical	34.8	0.721	244.0
Emergency & Critical Care Medicine	Medical	33.2	0.857	139.7
Endocrinology & Metabolism	Medical	37.9	0.746	186.0
Environmental & Occupational Health	Medical	37.2	0.602	187.5
Epidemiology	Medical	36.7	0.479	175.7
Gastroenterology & Hepatology	Medical	35.6	0.861	177.3
General & Internal Medicine	Medical	31.9	0.696	212.3
General Clinical Medicine	Medical	36.1	0.840	150.0
Genetics & Heredity	Medical	37.9	0.566	201.7
Geriatrics	Medical	37.8	0.723	215.2
Gerontology	Medical	36.1	0.539	240.0
Health Policy & Services	Medical	33.6	0.577	211.6
Immunology	Medical	36.1	0.780	190.9
Legal & Forensic Medicine	Medical	32.6	0.749	96.6
Microbiology	Medical	36.6	0.672	176.3
Microscopy	Medical	39.3	0.458	179.8
Mycology & Parasitology	Medical	38.5	0.653	115.1
Neurology & Neurosurgery	Medical	36.7	0.771	202.5
Nuclear Medicine & Medical Imaging	Medical	34.9	0.863	224.0
Nursing	Medical	33.8	0.717	206.6
Nutrition & Dietetics	Medical	37.7	0.568	170.5
Obstetrics & Reproductive Medicine	Medical	37.4	0.837	169.3
Oncology & Carcinogenesis	Medical	34.9	0.783	174.8
Ophthalmology & Optometry	Medical	38.6	0.852	167.2
Orthopedics	Medical	34.4	0.896	139.9
Otorhinolaryngology	Medical	36.3	0.875	173.3
Pathology	Medical	35.4	0.928	180.1
Pediatrics	Medical	36.2	0.773	137.1
Pharmacology & Pharmacy	Medical	35.3	0.704	151.8
Physiology	Medical	39.0	0.587	187.2
Psychiatry	Medical	37.0	0.745	192.3
Public Health	Medical	34.5	0.549	199.2
Rehabilitation	Medical	35.4	0.629	175.1
Respiratory System	Medical	36.7	0.844	187.2
Speech-Language Pathology & Audiology	Medical	35.8	0.611	212.6
Sport Sciences	Medical	32.2	0.728	179.2
Substance Abuse	Medical	36.9	0.591	159.5
Surgery	Medical	34.8	0.927	161.6
Toxicology	Medical	38.7	0.566	135.8
Tropical Medicine	Medical	35.1	0.563	133.9
Urology & Nephrology	Medical	36.2	0.885	172.4
Virology	Medical	36.9	0.690	175.5
Accounting	Professional	31.6	0.944	171.9

Architecture	Professional	31.9	0.576	202.6
Building & Construction	Professional	32.0	0.595	286.1
Business & Management	Professional	32.5	0.794	128.2
Design Practice & Management	Professional	31.5	0.494	239.0
Development Studies	Professional	33.6	0.621	192.0
Finance	Professional	32.8	0.934	98.5
Industrial Relations	Professional	33.2	0.756	75.2
Logistics & Transportation	Professional	31.1	0.666	224.8
Marketing	Professional	32.0	0.836	102.6
Sport, Leisure & Tourism	Professional	28.2	0.805	119.3
Urban & Regional Planning	Professional	35.5	0.564	320.0
Agricultural Economics & Policy	Social Sciences	34.7	0.649	295.2
Behavioral Science & Comparative Psychology	Social Sciences	39.1	0.570	232.4
Clinical Psychology	Social Sciences	35.3	0.671	187.6
Criminology	Social Sciences	35.1	0.704	188.0
Cultural Studies	Social Sciences	33.8	0.700	312.3
Demography	Social Sciences	39.9	0.625	256.6
Developmental & Child Psychology	Social Sciences	36.7	0.637	223.9
Econometrics	Social Sciences	33.6	0.849	290.1
Economic Theory	Social Sciences	42.8	0.778	284.3
Economics	Social Sciences	34.1	0.831	253.2
Education	Social Sciences	33.2	0.781	249.6
Experimental Psychology	Social Sciences	36.5	0.698	257.6
Family Studies	Social Sciences	36.0	0.637	233.3
Gender Studies	Social Sciences	38.1	0.624	207.0
General Psychology & Cognitive Sciences	Social Sciences	32.8	0.758	220.4
Geography	Social Sciences	33.0	0.608	280.0
History of Social Sciences	Social Sciences	39.7	0.536	313.8
Human Factors	Social Sciences	30.3	0.583	270.4
Information & Library Sciences	Social Sciences	28.1	0.714	223.7
International Relations	Social Sciences	34.5	0.733	221.0
Law	Social Sciences	26.6	0.797	160.6
Political Science & Public Administration	Social Sciences	33.7	0.775	293.9
Psychoanalysis	Social Sciences	42.1	0.883	132.6
Science Studies	Social Sciences	39.9	0.511	279.4
Social Psychology	Social Sciences	36.4	0.729	236.9
Social Sciences Methods	Social Sciences	37.7	0.566	262.1
Social Work	Social Sciences	30.4	0.673	251.3
Sociology	Social Sciences	34.5	0.718	325.7
Acoustics	STEM	37.2	0.581	228.6
Aerospace & Aeronautics	STEM	35.0	0.723	186.6
Agronomy & Agriculture	STEM	35.2	0.617	175.6
Analytical Chemistry	STEM	35.6	0.599	189.9
Applied Mathematics	STEM	37.4	0.616	252.6
Applied Physics	STEM	36.6	0.714	205.4
Artificial Intelligence & Image Processing	STEM	29.1	0.750	200.4
Astronomy & Astrophysics	STEM	33.5	0.896	201.0
Automobile Design & Engineering	STEM	40.0	0.929	1.0
Bioinformatics	STEM	31.3	0.427	223.1
Biomedical Engineering	STEM	33.2	0.493	256.3
Biotechnology	STEM	32.9	0.472	189.6
Chemical Engineering	STEM	36.6	0.570	191.1
Chemical Physics	STEM	39.1	0.598	216.7

Civil Engineering	STEM	35.1	0.658	234.2
Computation Theory & Mathematics	STEM	34.1	0.834	277.6
Computer Hardware & Architecture	STEM	30.6	0.766	290.4
Dairy & Animal Science	STEM	37.7	0.746	198.6
Distributed Computing	STEM	32.1	0.842	199.4
Ecology	STEM	34.3	0.734	217.2
Electrical & Electronic Engineering	STEM	29.3	0.710	179.9
Energy	STEM	34.5	0.646	169.7
Entomology	STEM	36.7	0.723	190.2
Environmental Engineering	STEM	36.1	0.592	218.1
Environmental Sciences	STEM	33.5	0.530	175.2
Evolutionary Biology	STEM	33.3	0.710	247.2
Fisheries	STEM	36.7	0.579	140.7
Fluids & Plasmas	STEM	39.9	0.732	217.4
Food Science	STEM	35.7	0.628	214.6
Forestry	STEM	32.4	0.562	144.2
General Chemistry	STEM	32.7	0.656	269.7
General Mathematics	STEM	39.0	0.837	267.6
General Physics	STEM	37.8	0.882	217.0
Geochemistry & Geophysics	STEM	38.8	0.845	209.8
Geological & Geomatics Engineering	STEM	35.2	0.630	210.6
Geology	STEM	40.5	0.875	142.2
Horticulture	STEM	31.4	0.628	174.0
Industrial Engineering & Automation	STEM	32.0	0.718	252.6
Information Systems	STEM	31.5	0.769	146.3
Inorganic & Nuclear Chemistry	STEM	39.8	0.755	183.5
Marine Biology & Hydrobiology	STEM	39.1	0.659	115.0
Materials	STEM	36.1	0.626	193.1
Mathematical Physics	STEM	44.4	0.779	261.1
Mechanical Engineering & Transports	STEM	38.3	0.646	199.4
Medical Informatics	STEM	32.4	0.588	213.5
Medicinal & Biomolecular Chemistry	STEM	34.2	0.605	102.4
Meteorology & Atmospheric Sciences	STEM	36.9	0.788	191.7
Mining & Metallurgy	STEM	40.2	0.705	142.1
Nanoscience & Nanotechnology	STEM	25.2	0.495	265.4
Networking & Telecommunications	STEM	29.9	0.805	218.5
Nuclear & Particle Physics	STEM	36.5	0.968	213.9
Numerical & Computational Mathematics	STEM	39.1	0.734	201.5
Oceanography	STEM	40.3	0.740	140.5
Operations Research	STEM	36.4	0.671	189.6
Optics	STEM	30.4	0.580	212.0
Optoelectronics & Photonics	STEM	34.9	0.552	132.0
Organic Chemistry	STEM	36.2	0.794	220.4
Ornithology	STEM	36.3	0.775	169.0
Paleontology	STEM	38.1	0.754	190.2
Physical Chemistry	STEM	34.4	0.524	171.3
Plant Biology & Botany	STEM	34.6	0.651	232.1
Polymers	STEM	36.6	0.595	196.9
Software Engineering	STEM	30.6	0.846	239.5
Statistics & Probability	STEM	35.6	0.566	301.0
Strategic, Defence & Security Studies	STEM	34.1	0.508	172.3
Veterinary Sciences	STEM	35.9	0.744	218.3
Zoology	STEM	35.0	0.795	111.4
